# 181. COVID-19 Infection is a Key Factor in Mortality for Staphylococcus aureus and Candida sp. Bloodstream Infections: an Evaluation of Outcomes before and during the COVID-19 Pandemic

**DOI:** 10.1093/ofid/ofad500.254

**Published:** 2023-11-27

**Authors:** Reese Cosimi, Florian Daragjati, Subhangi Ghosh, Ana Cristina Perez Moreno, Collin Miller, Karl Saake, Mohamad G Fakih

**Affiliations:** Ascension, Indianapolis, Indiana; Ascension, Indianapolis, Indiana; Ascension, Indianapolis, Indiana; Ascension Health, Franklin, Wisconsin; Ascension Health, Franklin, Wisconsin; Ascension, Indianapolis, Indiana; Ascension, Indianapolis, Indiana

## Abstract

**Background:**

*Staphylococcus aureus* and *Candida* sp. bloodstream infections (BSI) are associated with considerable mortality. The COVID-19 pandemic presented new challenges in the acute care setting which may affect outcomes in high-risk populations.

**Methods:**

Retrospective cross-sectional analysis across a large healthcare system of all admitted patients aged 18 years or older with *S. aureus* or *Candida* sp. BSI between pre-pandemic (January 2017-February 2020) and pandemic (March 2020- February 2023). The primary clinical outcome was all-cause mortality. Secondary outcomes include length of stay (LOS) and 30-day readmission. Data was stratified across the pre-pandemic and pandemic period, COVID-19 positive and negative patients, and community and hospital-onset BSI.

**Results:**

A total of 17,730 patients were included in the analysis. Baseline characteristics were similar between pre-pandemic and pandemic groups aside from higher hospital-onset infection rates in the pandemic COVID-19 positive population [Table 1]. No significant differences were found in mortality between COVID-19 negative groups [Table 2]. Concomitant COVID-19 infection was associated with increased mortality in both the *S. aureus* and *Candida* sp. BSI patients.

**Figure:**
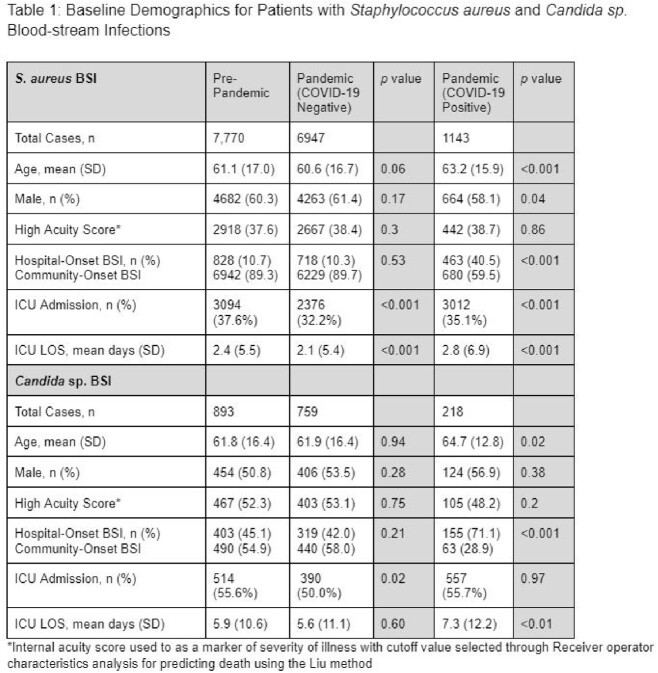

**Figure:**
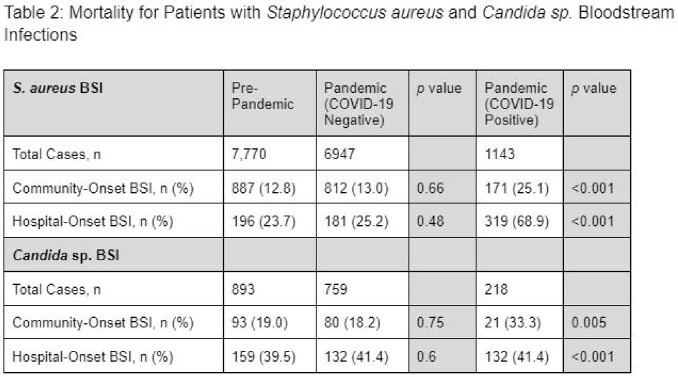

**Conclusion:**

While patients with concomitant COVID-19 infection had a substantially higher mortality compared to those without infection, the mortality in patients with *S. aureus* and *Candida* sp. community-onset and hospital-onset BSI did not change during the pandemic compared to pre-pandemic for patients without concomitant COVID-19 infection.

**Disclosures:**

**Reese Cosimi, PharmD**, Allergen: Advisor/Consultant

